# 2-Fluoro­anilinium *N*-(2-fluoro­phenyl)oxamate

**DOI:** 10.1107/S1600536808008891

**Published:** 2008-04-04

**Authors:** Orhan Büyükgüngör, Mustafa Odabaşoğlu

**Affiliations:** a Department of Physics, Faculty of Arts & Sciences, Ondokuz Mayıs University, TR-55139 Kurupelit Samsun, Turkey; b Department of Chemistry, Faculty of Arts & Sciences, Ondokuz Mayıs University, TR-55139 Kurupelit Samsun, Turkey

## Abstract

The crystal structure of the title salt, C_6_H_7_FN^+^·C_8_H_5_FNO_3_^−^, exhibits intra­molecular N—H⋯O and C—H⋯O and inter­molecular N—H⋯O and N—H⋯F hydrogen-bond inter­actions, the intra­molecular hydrogen-bond inter­actions generating *S*(6) and *S*(5) ring motifs. The dihedral angles between the aromatic ring and the intra­molecular hydrogen-bonded rings in the anion are 2.97 (7) and 6.70 (5)°. The two aromatic rings of the title compound are oriented with a dihedral angle of 77.25 (9)°.

## Related literature

For related structures see: Odabaşoğlu & Büyükgüngör (2006**a* ▶,*b* ▶,c* ▶); Büyükgüngör & Odabaşoğlu (2007 ▶). For ring motif details, see: Bernstein *et al.* (1995 ▶); Etter (1990 ▶).
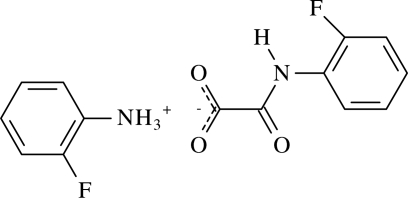


## Experimental

### 

#### Crystal data

C_6_H_7_FN^+^·C_8_H_5_FNO_3_^−^
*M* *_r_* = 294.26
Triclinic, 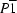

*a* = 6.7118 (9) Å
*b* = 9.5998 (14) Å
*c* = 11.7000 (16) Å
α = 68.346 (11)°
β = 85.791 (11)°
γ = 77.375 (11)°
*V* = 683.69 (18) Å^3^
*Z* = 2
Mo *K*α radiation
μ = 0.12 mm^−1^
*T* = 296 K
0.78 × 0.47 × 0.09 mm

#### Data collection

Stoe IPDSII diffractometer
Absorption correction: integration (*X-RED32*; Stoe & Cie, 2002 ▶) *T*_min_ = 0.931, *T*_max_ = 0.988
9218 measured reflections
2684 independent reflections
2023 reflections with *I* > 2σ(*I*)
*R*_int_ = 0.040

#### Refinement

*R*[*F* ^2^ > 2σ(*F* ^2^)] = 0.053
*wR*(*F* ^2^) = 0.168
*S* = 0.81
2684 reflections
203 parameters
H atoms treated by a mixture of independent and constrained refinement
Δρ_max_ = 0.53 e Å^−3^
Δρ_min_ = −0.30 e Å^−3^

### 

Data collection: *X-AREA* (Stoe & Cie, 2002 ▶); cell refinement: *X-AREA*; data reduction: *X-RED32* (Stoe & Cie, 2002 ▶); program(s) used to solve structure: *SHELXS97* (Sheldrick, 2008 ▶); program(s) used to refine structure: *SHELXL97* (Sheldrick, 2008 ▶); molecular graphics: *ORTEP-3 for Windows* (Farrugia, 1997 ▶); software used to prepare material for publication: *WinGX* (Farrugia, 1999 ▶).

## Supplementary Material

Crystal structure: contains datablocks I, global. DOI: 10.1107/S1600536808008891/at2556sup1.cif

Structure factors: contains datablocks I. DOI: 10.1107/S1600536808008891/at2556Isup2.hkl

Additional supplementary materials:  crystallographic information; 3D view; checkCIF report

## Figures and Tables

**Table 1:** Hydrogen-bond geometry (Å, °)

*D*—H⋯*A*	*D*—H	H⋯*A*	*D*⋯*A*	*D*—H⋯*A*
N1—H1⋯O3	0.86	2.19	2.623 (3)	111
N1—H1⋯F1^i^	0.86	2.61	3.313 (3)	140
N2—H2*A*⋯O2^ii^	0.93 (3)	1.77 (3)	2.696 (3)	170 (3)
N2—H2*B*⋯O3^iii^	0.90 (3)	1.86 (3)	2.753 (3)	174 (3)
N2—H2*C*⋯O2^iv^	0.89 (3)	2.05 (3)	2.802 (3)	142 (3)
N2—H2*C*⋯O1^iv^	0.89 (3)	2.30 (3)	3.041 (3)	141 (3)
C6—H6⋯O1	0.93	2.36	2.941 (3)	120

Symmetry codes: (i) 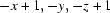; (ii) 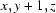; (iii) 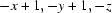; (iv) 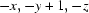.

**Fig. 1.**
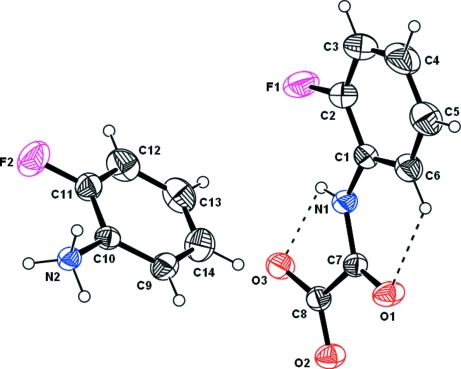
The molecular structure of the title molecule, with the atom-numbering scheme. Displacement ellipsoids are drawn at the 30% probability level. The hydrogen bonds are drawn as dashed lines.

**Fig. 2.**
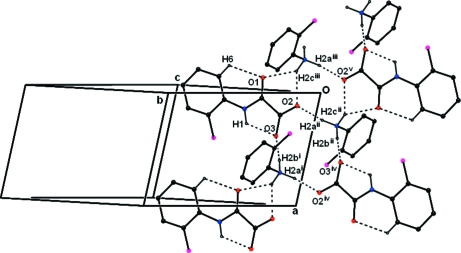
A partial packing diagram of (I), showing the formation of R12(5)R42(8)R44(12) motifs. H atoms not involved in hydrogen bonds have been omitted for clarity. [Symmetry codes: (i) 1 - x, 1 - y, z; (ii) x, y - 1, z; (iii) -x, y, -z; (iv) 1 - x, -y, -z; (v) -x, -y, -z].

**Fig. 3.**
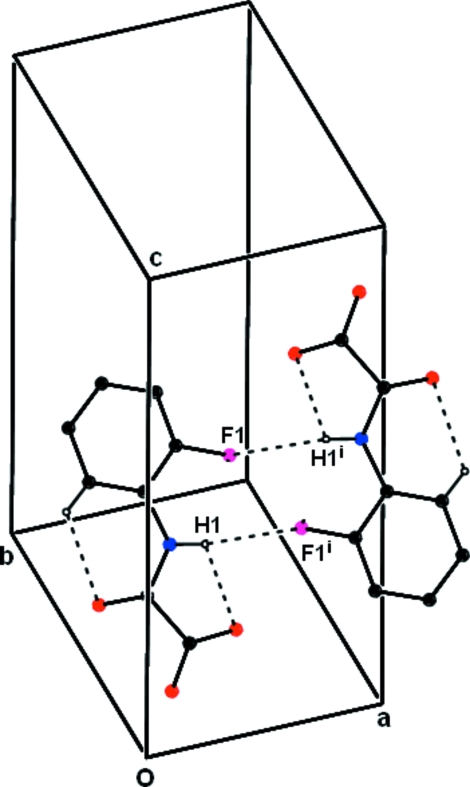
A partial packing diagram of (I), showing the formation of R22(10) motif. H atoms not involved in hydrogen bonds have been omitted for clarity. [Symmetry code: (i) 1 - x, -y, 1 - z].

## supplementary crystallographic information

### Comment

The present work is part of a structural study of compounds of anillinium carboxylates and we report here the structure of 2-fluoroanilinium 2-(2-fluorophenylamino)-2-oxoacetate, (I).

The title compound (I) is built up from a 2-fluoroanilinium cation and 2-(2-fluorophenylamino)-2-oxoacetate anion (Fig. 1). There are two intramolecular hydrogen bonds which generate S(6)and S(5) motifs (Bernstein *et al.*, 1995; Etter, 1990), and the anions and cations of (I) are linked to each other by five intermolecular hydrogen bond interactions (Table 1.). The intermolecular hydrogen interactions, except N—H···F, generate *R*_1_^2^(5)*R*_4_^2^(8)*R*_4_^4^(12) motifs (Fig. 2) and these motifs link N—H···F hydrogen bonded *R*_2_^2^(10) rings (Fig. 3) forming a three dimensional network. The dihedral angles between the aromatic ring and intramolecular hydrogen bonded rings in the anion are 2.97 (7)° and 6.70 (5)°. The two aromatic rings of the title compound are oriented with a dihedral angle of 77.25 (9)°.

### Experimental

The title compound was prepared according to the method described by Büyükgüngör & Odabaşoǧlu (2006*b*), using 2-fluoroaniline and oxalic acid as starting materials (yield 90%). Crystals of (I) suitable for *x*-ray analysis were obtained by slow evaporation of an ethanol (95%) solution at room temperature.

### Refinement

H atom (for NH_3_) was located in difference synthesis and refined freely. The remaining H atoms were positioned geometrically, with C—H = 0.93 for aromatic H and N—H = 0.86 Å for amine H, and constrained to ride on their parent atoms with *U*_iso_(H) = 1.2*U*_eq_(parent).

### Crystal data

**Table:**

C_6_H_7_FN^+^·C_8_H_5_FNO_3_^–^	*Z* = 2
*M**_r_* = 294.26	*F*_000_ = 304
Triclinic, *P*1	*D*_x_ = 1.429 Mg m^−^^3^
Hall symbol: -P 1	Mo *K*α radiation λ = 0.71073 Å
*a* = 6.7118 (9) Å	Cell parameters from 9218 reflections
*b* = 9.5998 (14) Å	θ = 1.9–28.1º
*c* = 11.7000 (16) Å	µ = 0.12 mm^−^^1^
α = 68.346 (11)º	*T* = 296 K
β = 85.791 (11)º	Plate, colourless
γ = 77.375 (11)º	0.78 × 0.47 × 0.09 mm
*V* = 683.69 (18) Å^3^	

### Data collection

**Table:**

Stoe IPDSII diffractometer	2684 independent reflections
Monochromator: plane graphite	2023 reflections with *I* > 2σ(*I*)
Detector resolution: 6.67 pixels mm^-1^	*R*_int_ = 0.040
*T* = 296 K	θ_max_ = 26.0º
ω rotation method scans	θ_min_ = 1.9º
Absorption correction: integration(X-RED32; Stoe & Cie, 2002)	*h* = −8→8
*T*_min_ = 0.932, *T*_max_ = 0.988	*k* = −11→11
9218 measured reflections	*l* = −14→14

### Refinement

**Table:**

Refinement on *F*^2^	Hydrogen site location: inferred from neighbouring sites
Least-squares matrix: full	H atoms treated by a mixture of independent and constrained refinement
*R*[*F*^2^ > 2σ(*F*^2^)] = 0.053	*w* = 1/[σ^2^(*F*_o_^2^) + (0.1051*P*)^2^ + 0.5375*P*] where *P* = (*F*_o_^2^ + 2*F*_c_^2^)/3
*wR*(*F*^2^) = 0.168	(Δ/σ)_max_ < 0.001
*S* = 0.81	Δρ_max_ = 0.53 e Å^−^^3^
2684 reflections	Δρ_min_ = −0.30 e Å^−^^3^
203 parameters	Extinction correction: SHELXL97 (Sheldrick, 2008), Fc^*^=kFc[1+0.001xFc^2^λ^3^/sin(2θ)]^-1/4^
Primary atom site location: structure-invariant direct methods	Extinction coefficient: 0.026 (7)
Secondary atom site location: difference Fourier map	

### Special details

**Table:**

Geometry. All e.s.d.'s (except the e.s.d. in the dihedral angle between two l.s. planes) are estimated using the full covariance matrix. The cell e.s.d.'s are taken into account individually in the estimation of e.s.d.'s in distances, angles and torsion angles; correlations between e.s.d.'s in cell parameters are only used when they are defined by crystal symmetry. An approximate (isotropic) treatment of cell e.s.d.'s is used for estimating e.s.d.'s involving l.s. planes.
Refinement. Refinement of *F*^2^ against ALL reflections. The weighted *R*-factor *wR* and goodness of fit *S* are based on *F*^2^, conventional *R*-factors *R* are based on *F*, with *F* set to zero for negative *F*^2^. The threshold expression of *F*^2^ > σ(*F*^2^) is used only for calculating *R*-factors(gt) *etc*. and is not relevant to the choice of reflections for refinement. *R*-factors based on *F*^2^ are statistically about twice as large as those based on *F*, and *R*- factors based on ALL data will be even larger.

### Fractional atomic coordinates and isotropic or equivalent isotropic displacement parameters (Å^2^)

**Table:**

	*x*	*y*	*z*	*U*_iso_*/*U*_eq_	
C1	0.0927 (4)	0.1895 (3)	0.4566 (2)	0.0500 (6)	
C2	0.2329 (5)	0.2000 (4)	0.5327 (3)	0.0662 (7)	
C3	0.1793 (7)	0.2559 (5)	0.6239 (3)	0.0882 (10)	
H3	0.2783	0.2599	0.6735	0.106*	
C4	−0.0226 (7)	0.3060 (5)	0.6414 (3)	0.0958 (12)	
H4	−0.0625	0.3456	0.7030	0.115*	
C5	−0.1675 (6)	0.2980 (4)	0.5682 (3)	0.0826 (9)	
H5	−0.3050	0.3322	0.5809	0.099*	
C6	−0.1122 (4)	0.2399 (3)	0.4759 (2)	0.0619 (7)	
H6	−0.2116	0.2347	0.4272	0.074*	
C7	0.0672 (3)	0.1176 (3)	0.2757 (2)	0.0452 (5)	
C8	0.2108 (3)	0.0541 (3)	0.1899 (2)	0.0472 (5)	
C9	0.5179 (4)	0.6758 (3)	0.1045 (2)	0.0592 (7)	
C10	0.3396 (3)	0.7306 (3)	0.0369 (2)	0.0447 (5)	
C11	0.2088 (5)	0.6328 (3)	0.0524 (3)	0.0625 (7)	
H11	0.0855	0.6678	0.0095	0.075*	
C12	0.2612 (6)	0.4827 (4)	0.1318 (3)	0.0804 (9)	
H12	0.1723	0.4171	0.1420	0.097*	
C13	0.4415 (6)	0.4291 (4)	0.1955 (3)	0.0803 (9)	
H13	0.4763	0.3270	0.2472	0.096*	
C14	0.5722 (5)	0.5263 (4)	0.1834 (3)	0.0787 (9)	
H14	0.6942	0.4915	0.2277	0.094*	
N1	0.1682 (3)	0.1288 (2)	0.36637 (17)	0.0496 (5)	
H1	0.2978	0.0935	0.3692	0.060*	
N2	0.2905 (3)	0.8889 (2)	−0.04487 (19)	0.0447 (5)	
O1	−0.1162 (3)	0.1563 (2)	0.25841 (17)	0.0638 (5)	
O2	0.1240 (3)	0.0321 (2)	0.10956 (16)	0.0587 (5)	
O3	0.3972 (3)	0.0349 (2)	0.20670 (16)	0.0651 (5)	
F1	0.4339 (3)	0.1515 (3)	0.51255 (18)	0.0919 (7)	
F2	0.6443 (3)	0.7710 (2)	0.0900 (2)	0.1002 (7)	
H2A	0.243 (5)	0.946 (3)	0.004 (3)	0.069 (8)*	
H2B	0.396 (5)	0.915 (3)	−0.094 (3)	0.064 (8)*	
H2C	0.187 (5)	0.898 (3)	−0.092 (3)	0.073 (9)*	

### Atomic displacement parameters (Å^2^)

**Table:**

	*U*^11^	*U*^22^	*U*^33^	*U*^12^	*U*^13^	*U*^23^
C1	0.0559 (14)	0.0517 (13)	0.0407 (12)	−0.0165 (11)	0.0003 (10)	−0.0118 (10)
C2	0.0678 (18)	0.0816 (19)	0.0546 (15)	−0.0240 (15)	−0.0014 (13)	−0.0257 (14)
C3	0.107 (3)	0.115 (3)	0.0628 (19)	−0.040 (2)	0.0018 (18)	−0.0461 (19)
C4	0.134 (4)	0.105 (3)	0.068 (2)	−0.035 (2)	0.020 (2)	−0.051 (2)
C5	0.086 (2)	0.090 (2)	0.075 (2)	−0.0135 (18)	0.0180 (17)	−0.0395 (18)
C6	0.0621 (16)	0.0686 (17)	0.0554 (15)	−0.0137 (13)	0.0054 (12)	−0.0239 (13)
C7	0.0433 (13)	0.0461 (12)	0.0438 (12)	−0.0093 (10)	−0.0035 (9)	−0.0128 (10)
C8	0.0418 (12)	0.0501 (13)	0.0448 (12)	−0.0068 (10)	−0.0029 (9)	−0.0124 (10)
C9	0.0528 (15)	0.0617 (16)	0.0590 (15)	−0.0124 (12)	−0.0016 (12)	−0.0166 (12)
C10	0.0465 (12)	0.0475 (12)	0.0420 (11)	−0.0057 (10)	0.0015 (9)	−0.0211 (10)
C11	0.0719 (18)	0.0591 (16)	0.0633 (16)	−0.0226 (13)	−0.0022 (13)	−0.0243 (13)
C12	0.102 (3)	0.0582 (18)	0.083 (2)	−0.0320 (17)	0.0009 (19)	−0.0194 (16)
C13	0.102 (3)	0.0503 (16)	0.0728 (19)	−0.0055 (16)	0.0036 (18)	−0.0103 (14)
C14	0.0662 (19)	0.078 (2)	0.0695 (19)	0.0046 (16)	−0.0092 (15)	−0.0104 (16)
N1	0.0409 (10)	0.0623 (12)	0.0463 (11)	−0.0105 (9)	−0.0033 (8)	−0.0198 (9)
N2	0.0391 (11)	0.0510 (11)	0.0458 (11)	−0.0096 (8)	−0.0009 (9)	−0.0192 (9)
O1	0.0404 (10)	0.0909 (14)	0.0671 (11)	−0.0012 (9)	−0.0080 (8)	−0.0420 (10)
O2	0.0498 (10)	0.0734 (12)	0.0605 (10)	−0.0046 (8)	−0.0034 (8)	−0.0369 (9)
O3	0.0402 (10)	0.0987 (15)	0.0522 (10)	−0.0088 (9)	−0.0004 (7)	−0.0254 (10)
F1	0.0653 (11)	0.1443 (18)	0.0815 (12)	−0.0250 (11)	−0.0134 (9)	−0.0540 (12)
F2	0.0742 (12)	0.0985 (15)	0.1081 (15)	−0.0317 (10)	−0.0328 (11)	−0.0010 (12)

### Geometric parameters (Å, °)

**Table:**

C1—C6	1.386 (4)	C9—F2	1.337 (3)
C1—C2	1.385 (4)	C9—C14	1.374 (4)
C1—N1	1.399 (3)	C9—C10	1.375 (3)
C2—C3	1.356 (5)	C10—C11	1.377 (3)
C2—F1	1.361 (4)	C10—N2	1.445 (3)
C3—C4	1.363 (5)	C11—C12	1.378 (4)
C3—H3	0.9300	C11—H11	0.9300
C4—C5	1.375 (5)	C12—C13	1.365 (5)
C4—H4	0.9300	C12—H12	0.9300
C5—C6	1.381 (4)	C13—C14	1.379 (5)
C5—H5	0.9300	C13—H13	0.9300
C6—H6	0.9300	C14—H14	0.9300
C7—O1	1.214 (3)	N1—H1	0.8600
C7—N1	1.349 (3)	N2—H2A	0.93 (3)
C7—C8	1.536 (3)	N2—H2B	0.90 (3)
C8—O3	1.244 (3)	N2—H2C	0.89 (3)
C8—O2	1.244 (3)		
			
C6—C1—C2	117.3 (2)	C14—C9—C10	122.3 (3)
C6—C1—N1	125.0 (2)	C9—C10—C11	118.4 (2)
C2—C1—N1	117.7 (2)	C9—C10—N2	119.9 (2)
C3—C2—F1	119.5 (3)	C11—C10—N2	121.7 (2)
C3—C2—C1	123.4 (3)	C12—C11—C10	119.8 (3)
F1—C2—C1	117.0 (2)	C12—C11—H11	120.1
C2—C3—C4	118.7 (3)	C10—C11—H11	120.1
C2—C3—H3	120.7	C13—C12—C11	120.9 (3)
C4—C3—H3	120.7	C13—C12—H12	119.5
C3—C4—C5	120.0 (3)	C11—C12—H12	119.5
C3—C4—H4	120.0	C12—C13—C14	120.1 (3)
C5—C4—H4	120.0	C12—C13—H13	120.0
C6—C5—C4	121.1 (3)	C14—C13—H13	120.0
C6—C5—H5	119.5	C9—C14—C13	118.5 (3)
C4—C5—H5	119.5	C9—C14—H14	120.8
C5—C6—C1	119.5 (3)	C13—C14—H14	120.8
C5—C6—H6	120.3	C7—N1—C1	129.4 (2)
C1—C6—H6	120.3	C7—N1—H1	115.3
O1—C7—N1	125.5 (2)	C1—N1—H1	115.3
O1—C7—C8	121.8 (2)	C10—N2—H2A	106.5 (18)
N1—C7—C8	112.65 (19)	C10—N2—H2B	111.4 (18)
O3—C8—O2	128.1 (2)	H2A—N2—H2B	116 (3)
O3—C8—C7	116.8 (2)	C10—N2—H2C	106.7 (19)
O2—C8—C7	115.1 (2)	H2A—N2—H2C	108 (3)
F2—C9—C14	119.3 (3)	H2B—N2—H2C	108 (3)
F2—C9—C10	118.4 (2)		
			
C6—C1—C2—C3	0.2 (4)	F2—C9—C10—C11	−179.6 (2)
N1—C1—C2—C3	−179.6 (3)	C14—C9—C10—C11	2.2 (4)
C6—C1—C2—F1	−179.5 (2)	F2—C9—C10—N2	−1.7 (4)
N1—C1—C2—F1	0.7 (4)	C14—C9—C10—N2	−179.9 (3)
F1—C2—C3—C4	179.0 (3)	C9—C10—C11—C12	−1.8 (4)
C1—C2—C3—C4	−0.7 (5)	N2—C10—C11—C12	−179.7 (3)
C2—C3—C4—C5	0.6 (6)	C10—C11—C12—C13	0.0 (5)
C3—C4—C5—C6	−0.2 (6)	C11—C12—C13—C14	1.6 (5)
C4—C5—C6—C1	−0.3 (5)	F2—C9—C14—C13	−178.8 (3)
C2—C1—C6—C5	0.2 (4)	C10—C9—C14—C13	−0.6 (5)
N1—C1—C6—C5	−179.9 (3)	C12—C13—C14—C9	−1.3 (5)
O1—C7—C8—O3	171.7 (2)	O1—C7—N1—C1	−2.4 (4)
N1—C7—C8—O3	−6.3 (3)	C8—C7—N1—C1	175.6 (2)
O1—C7—C8—O2	−6.5 (3)	C6—C1—N1—C7	6.0 (4)
N1—C7—C8—O2	175.5 (2)	C2—C1—N1—C7	−174.2 (2)

### Hydrogen-bond geometry (Å, °)

**Table:**

*D*—H···*A*	*D*—H	H···*A*	*D*···*A*	*D*—H···*A*
N1—H1···O3	0.86	2.19	2.623 (3)	111
N1—H1···F1^i^	0.86	2.61	3.313 (3)	140
N2—H2A···O2^ii^	0.93 (3)	1.77 (3)	2.696 (3)	170 (3)
N2—H2B···O3^iii^	0.90 (3)	1.86 (3)	2.753 (3)	174 (3)
N2—H2C···O2^iv^	0.89 (3)	2.05 (3)	2.802 (3)	142 (3)
N2—H2C···O1^iv^	0.89 (3)	2.30 (3)	3.041 (3)	141 (3)
C6—H6···O1	0.93	2.36	2.941 (3)	120

Symmetry codes: (i) −*x*+1, −*y*, −*z*+1; (ii) *x*, *y*+1, *z*; (iii) −*x*+1, −*y*+1, −*z*; (iv) −*x*, −*y*+1, −*z*.
